# Dataset on cost comparative analysis of different walling materials in residential buildings in a developing economy

**DOI:** 10.1016/j.dib.2018.06.102

**Published:** 2018-07-03

**Authors:** Bukola Adejoke Adewale, Olatunde Daniel Babalola, Foluke Oladunni Jegede, Adedeji Afolabi, Temilola Oyenuga, Chukwuma Obi

**Affiliations:** Covenant University, Nigeria

## Abstract

The walls which form the envelope of a building are very important, therefore, the choice of materials used for it is very important. The dataset presents a comparative analysis of different walling materials used in residential buildings in a developing economy. The data was presented using descriptive tools of figures and tables. The dataset was obtained within several parts in Lagos State, Nigeria. The dataset showed the awareness and rate of use of selected walling materials by home owners in residential apartments. Furthermore, a comparative analysis of the cost implication of using eight (8) different walling materials in a typical two (2)- bedroom residential building was carried out. Using a market survey armed with current prices, a bill of quantities showed the varying cost of different walling materials for residential buildings. The dataset when analyzed can help in understanding the dynamics of providing affordable housing in developing economies. In addition, prospective home owners and building materials’ investors can benefit from the dataset.

**Specifications Table**

**Table:** 

Subject area	*Construction*
More specific subject area	*Building materials*
Type of data	*Tables and figures*
How data was acquired	*Field survey and market survey*
Data format	*Raw*
Experimental factors	*Purposive Sampling of residential buildings*
Experimental features	*Comparative study of varying walling materials*
Data source location	*Lagos, Nigeria*
Data accessibility	*All data is presented*

**Value of the data**

• The dataset showed different walling materials that can used for residential buildings in developing countries.
• The dataset gives a basis for selection of walling materials according to the financial status of residents in a developing economy.
• The dataset is able to inform home owners/developers about the various walling materials available in the building materials market.
• A critical dissection of the data revealed that walling materials in the building materials׳ market is limited within the selected area. Therefore, the data would be relevant to investors in the building materials sector by providing alternative and affordable walling materials for housing in developing countries.
• The dataset would educate practicing architects on the varied types of walling materials and spur researchers in innovating new walling materials that can be used in this clime.

## 1 Data

**Fig. 1:**
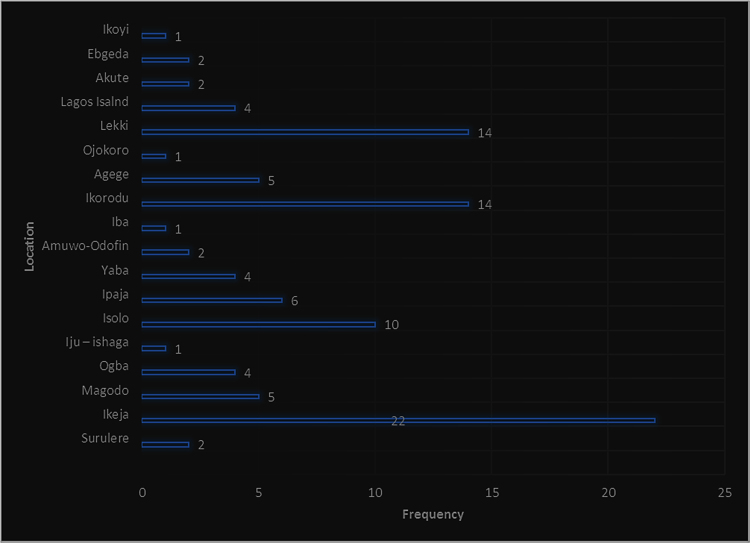
Surveyed residential areas in Lagos state, Nigeria.

**Fig. 2:**
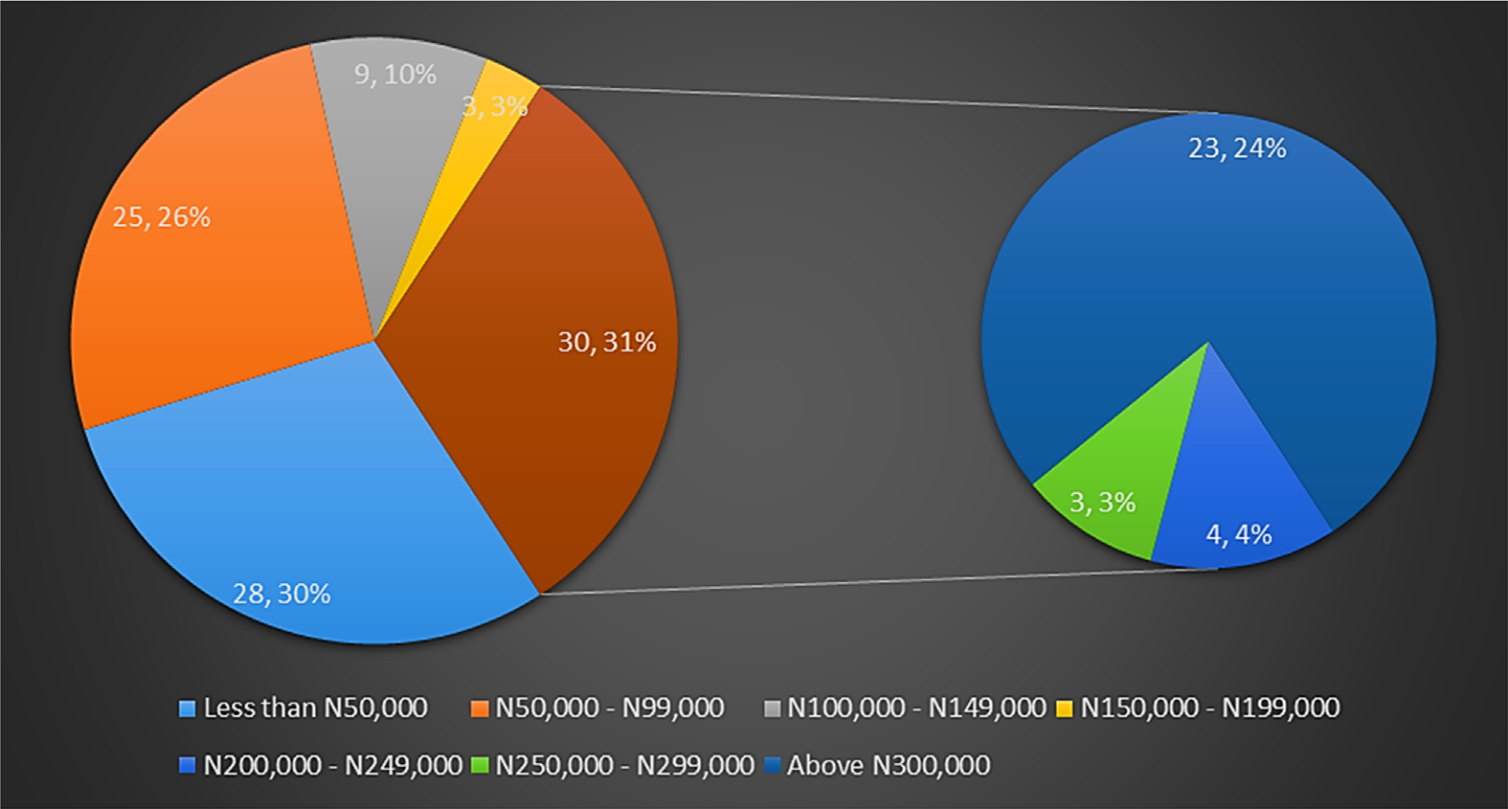
Monthly income stratification of residential homeowners in Lagos State.

**Fig. 3:**
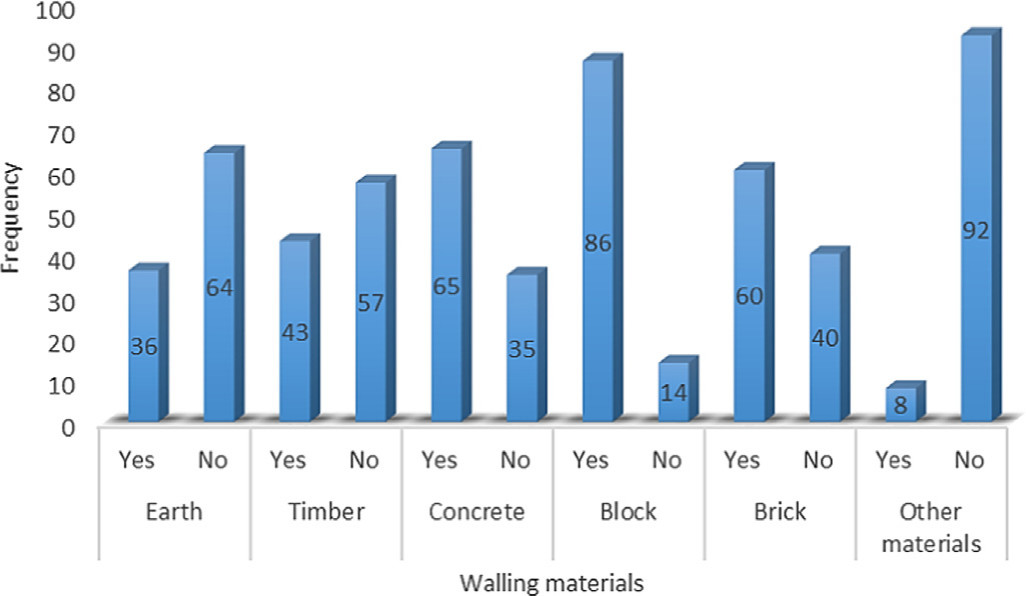
Awareness of selected walling materials.

**Fig. 4:**
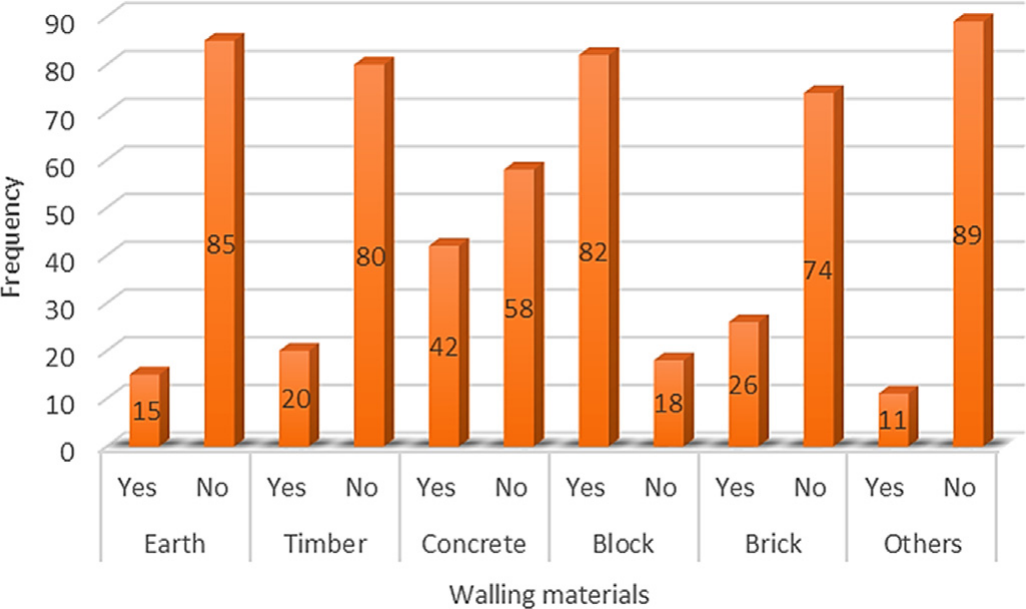
Walling materials used by homeowners.

**Fig. 5:**
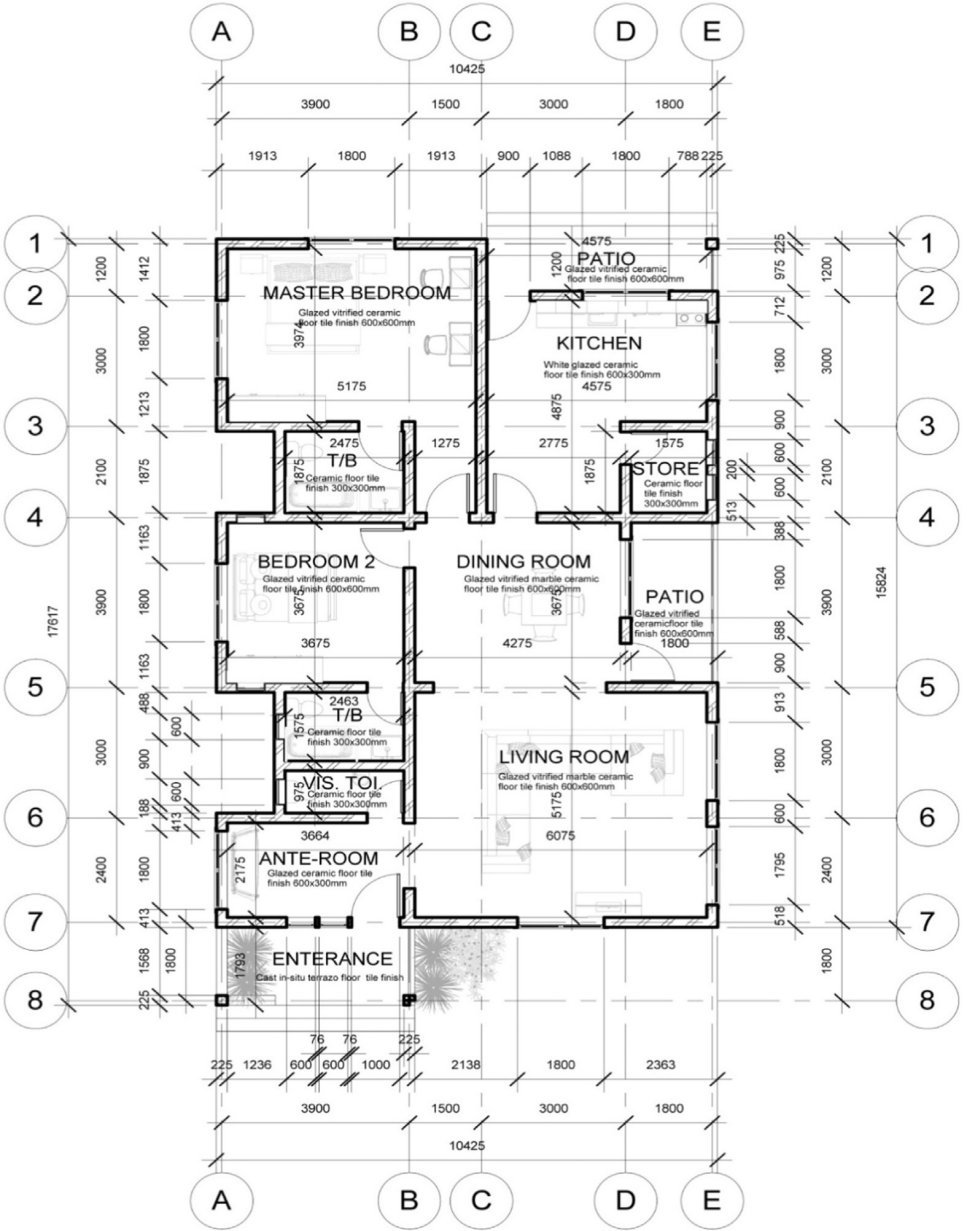
Floor plan of two-bedroom bungalow.

**Fig. 6:**
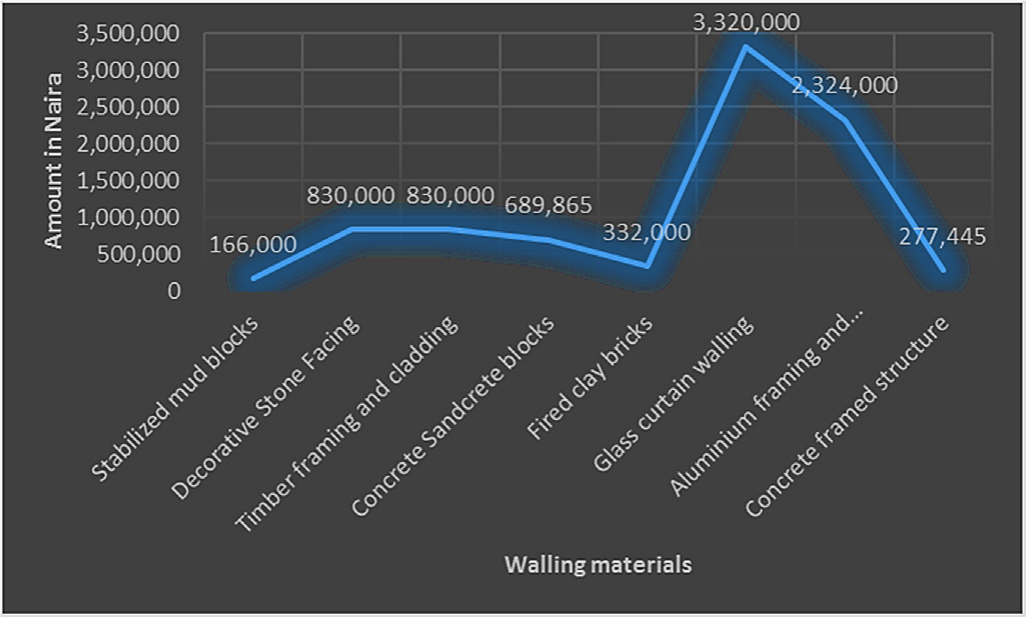
Cost comparison of selected walling materials for residential units.

In developing economies such as in Nigeria, people use different types of walling materials for their homes majorly for three probable reasons – cost of material, durability and aesthetics [1], [2], [3], [4], [5]. These factors are what people basically base their selection of walling materials for their houses on. The dataset presented the angle of cost of materials. This becomes imperative as the issues of affordable housing in terms of quality and quantity poses a challenge in developing economies [6], [7]. The dataset measured the residents׳ awareness of walling materials and the walling materials that has been used by the home owners for their residential apartment. This aspect of the data was collected with a structured questionnaire from eighteen (18) locations within Lagos State, Nigeria. Fig. 1 shows the residential apartments visited in various location within the study area. The highest areas surveyed include Ikeja (22), Lekki (14), Ikorodu (14) and Isolo (10). A total of 71 residential apartments were surveyed. These areas are densely populated areas within Lagos. The monthly income breakdown of the home owners showed in Fig. 2 revealed that from the area surveyed 28 homeowners earned less than 50,000 naira, 25 homeowners earned between 50,000 and 99,000 naira, 9 homeowners earned between 100,000 and 149,000 naira, 3 home owners earned between 150,000 and 199,000 naira, 4 homeowners earned between 200,000 and 249,000 naira, 3 homeowners earned between 250,000 and 299,000 naira while 23 homeowners earned above 300,000 naira monthly. A review of the selected walling materials in Fig. 3 showed that homeowners were mostly aware in the use of blocks (86%), concrete (65%) and bricks (60%) for erecting walls in their residential units. The dataset revealed that there was limited knowledge in the use of earth (36%), timber (43%) and other walling materials (8%) for erecting walls in residential units. Fig. 4 shows walling materials that have been mostly used by homeowners in the area surveyed. In Fig. 4, blocks (82%) are mostly used for walling materials, while there is a limited use in the use of walling materials such as earth (15%), timber (20%), concrete (42%), brick (26%) and other materials (11%). In order to carry out the comparative analysis of the cost of selected walling materials for residential units, a typical two-bedroom floor plan was used as shown in Fig. 5. The cost price of the 166 m^2^ walling area was determined using a market survey of the unit price of eight (8) walling materials for residential units. Table 1 shows the bill of quantities (BOQ) prepared for the cost analysis which described the walling materials and the process of estimation for residential units. From Table 1, the cost comparison of the selected walling materials was carried out. Fig. 6 shows the cost comparison of eight (8) walling materials based on the market survey within the area surveyed. From Fig. 6, a breakdown of the cost showed that to erect an external walling system for a two-bedroom residential unit of 166 m^2^, the cost price of each walling material would be stabilized mud blocks (166,000 naira), decorative stone facing (830,000 naira), timber framing and cladding (830,000 naira), concrete sandcrete blocks (689,865 naira), fired clay bricks (332,000 naira), glass curtain walling (3,320,000 naira), aluminium framing (2,324,000 naira) and alucoboard cladding and concrete framed structure (277,445 naira). The dataset in Fig. 6 would help homeowners and developers of residential units in making affordable housing possible with preference to the income of potential homeowners. In addition, by observing the dataset, researchers and investors can introduce cheaper walling materials in the building materials’ market that can compete with the cost of the selected walling materials in this dataset.

**Table 1:** Bill of quantities for selected walling of two-bedroom bungalow.

**Item**	**Description**	**Qty**	**Unit**	**Rate**	**Amount(N)**
	**Option 1: Stabilized mud blocks**				
A	Stabilized mud blocks	166	m^2^	1000	**166,000.00**
	**Option 2: Decorative Stone Facing**				
B	Decorative non-load bearing wall; 75/100 mm thick overall, built against existing block wall from dressed granite boulder 150/200 mm maximum boulder size in cement mortar (1:3) and pointed with neat grooved joints as work proceeds	166	m^2^	5000	**830,000.00**
	**Option 3: Timber framing and cladding**				
C	Timber framing and PVC cladding on wall externally	166	m^2^	5000	**830,000.00**
	**Option 4: Concrete Sandcrete blocks**				
D	Vibrated hollow sandcrete blocks in cement mortar on wall 230 mm thick	166	m^2^	2800	464,800.00
E	Reinforced concrete (1:2:4 – 19 mm agg) in lintel	3	m^3^	24,000	72,000.00
F	12 mm diameter bar reinforcement in lintel	240	kg	235	56,400.00
G	10 mm diameter bar stirrups in lintel	179	kg	235	42,065.00
H	Sawn formwork to vertical sides of lintel	42	m^2^	1300	54,600.00
					**689,865.00**
	**Option 5: Fired clay bricks**				
I	Fired clay bricks	166	m^2^	2000	**332,000.00**
	**Option 6: Glass curtain walling**				
J	Pittsburgh corning “decora” or other equal approved block size 200 mm×200 mm×100 mm bedded and jointed in approved adhesives	166	m^2^	20,000	**3,320,000.00**
	**Option 7: Aluminium framing and alucoboard cladding**				
K	Aluminium framing and alucoboard cladding	166	m^2^	14,000	**2,324,000.00**
	**Option 8: Concrete framed structure**				
L	Reinforced concrete (1:2:4 – 19 mm agg) in column	4	m^3^	24,000	96,000.00
M	12 mm diameter bar reinforcement in column	256	kg	235	60,160.00
N	10 mm diameter bar stirrups in column	151	kg	235	35,485.00
O	Sawn formwork to vertical sides of column	66	m^2^	1300	85,800.00
					**277,445.00**

## 2 Experimental design, materials, and methods

Using a structured questionnaire, a total of one hundred (100) respondents participated in the dataset on cost comparison of walling materials for residential units in a developing economy. The questionnaires were purposively distributed to 100 household heads or their representatives in 71 housing units surveyed in 18 local government areas in Lagos State. Lagos State was selected for the dataset due to the pressing challenges of providing housing for its over 20 million citizens. The questionnaire was designed to elicit responses on the awareness of residents of varied walling materials and the rate of use of the walling materials. Other dataset on measuring perception of respondent that have been obtained in like manner can be found in Refs. [8], [9], [10], [11]. The dataset from the questionnaire were presented using figures. The descriptive dataset are in relation to other dataset in Refs. [12], [13], [14], [15]. Apart from the data gathered through questionnaires, a complementary investigation was conducted to know the cost of different walling materials in residential units. Market survey of these materials was done. The Bill of Quantities (BOQ) of the identified walling materials (sandcrete blocks, decorated stone facing, timber framing and cladding, stabilised mud blocks, fired clay bricks, glass curtain walling, aluminium frame and Aluco board cladding, and concrete framed structure) was prepared, using a typical two-bedroom bungalow as the basis of measurement.
