# Supplementary material for: Dataset on cost comparative analysis of different walling materials in residential buildings in a developing economy
Source: Data Brief. 2018 Jul 3;19:1918–24. doi: 10.1016/j.dib.2018.06.102 (PMC6141787; doi:10.1016/j.dib.2018.06.102)
Supplement: Supplementary file 1 — Supplementary material [file mmc1.docx]

Dear Editor,

We would like to bring to your notice that there are no interests to declare.

Bukola, Olatunde, Foluke, Temilola, Chukwuma
